# Advancing translational exposomics: bridging genome, exposome and personalized medicine

**DOI:** 10.1186/s40246-025-00761-6

**Published:** 2025-04-30

**Authors:** Dimosthenis Sarigiannis, Spyros Karakitsios, Ourania Anesti, Arthur Stem, Damaskini Valvi, Susan C.J. Sumner, Leda Chatzi, Michael P. Snyder, David C. Thompson, Vasilis Vasiliou

**Affiliations:** 1 National Hellenic Research Foundation, 48 Vassileos Constantinou Avenue, Athens, 11635 Greece; 2 Department of Chemical Engineering, Environmental Engineering Laboratory, Aristotle University of Thessaloniki, University Campus, Thessaloniki, 54124 Greece; 3 HERACLES Research Center on the Exposome and Health, Center for Interdisciplinary Research and Innovation, Aristotle University of Thessaloniki, Balkan Center, Bldg. B, 10th km Thessaloniki-Thermi Road, Thessaloniki, 57001 Greece; 4 University School for Advanced Study (IUSS), Science, Technology and Society Department, Environmental Health Engineering, Piazza della Vittoria 15, Pavia, 27100 Italy; 5 School of Medicine, University of Crete, Heraklion, Crete, 71500 Greece; 6 Department of Environmental Health Sciences, Yale School of Public Health, New Haven, CT 06510 USA; 7 Department of Environmental Medicine, Icahn School of Medicine at Mount Sinai, New York, NY 10029 USA; 8 Departments of Nutrition and Pharmacology, UNC Nutrition Research Institute, UNC Chapel Hill, Kannapolis, NC 28010 USA; 9 Department of Population and Public Health Sciences, University of Southern California Keck School of Medicine, Los Angeles, CA USA; 10 Department of Genetics, Stanford University School of Medicine, Stanford University, Stanford, CA USA

## Abstract

Understanding the interplay between genetic predisposition and environmental and lifestyle exposures is essential for advancing precision medicine and public health. The exposome, defined as the sum of all environmental exposures an individual encounters throughout their lifetime, complements genomic data by elucidating how external and internal exposure factors influence health outcomes. This treatise highlights the emerging discipline of translational exposomics that integrates exposomics and genomics, offering a comprehensive approach to decipher the complex relationships between environmental and lifestyle exposures, genetic variability, and disease phenotypes. We highlight cutting-edge methodologies, including multi-omics technologies, exposome-wide association studies (EWAS), physiology-based biokinetic modeling, and advanced bioinformatics approaches. These tools enable precise characterization of both the external and the internal exposome, facilitating the identification of biomarkers, exposure-response relationships, and disease prediction and mechanisms. We also consider the importance of addressing socio-economic, demographic, and gender disparities in environmental health research. We emphasize how exposome data can contextualize genomic variation and enhance causal inference, especially in studies of vulnerable populations and complex diseases. By showcasing concrete examples and proposing integrative platforms for translational exposomics, this work underscores the critical need to bridge genomics and exposomics to enable precision prevention, risk stratification, and public health decision-making. This integrative approach offers a new paradigm for understanding health and disease beyond genetics alone.

## Introduction

The exposome encompasses all environmental factors/exposures throughout life (from preconception to death) that influence health and disease [1]. This concept also includes multigenerational and transgenerational exposures—where environmental insults experienced by parents (or even grandparents) may impact the health of subsequent generations through epigenetic modifications, altered maternal physiology, or germline transmission of environmentally induced effects [2, 3]. Exposome research complements and builds upon genomic research. Although decoding the human genome [4] increased our understanding of the underlying causes of disease, the genome itself explains only a fraction of the burden of disease in the human population [5–7]. The contribution of environmental factors to health outcomes varies depending on the specific condition studied and can often rival or exceed that of genetic factors. For example, studies have estimated that environmental determinants contribute approximately 70–90% of the attributable risk in chronic diseases such as cardiovascular disease and certain cancers, compared to a smaller proportion explained by inherited genetic variation [5–8]. Thus, quantifying the relative impact of environmental and genetic risk factors is crucial for accurate disease modeling and prevention strategies [5–8].

Particularly critical is the interaction of environmental factors (e.g., chemical exposures, dietary, physical activity, medications, drugs, tobacco use, behavioral, and social choices) with biological systems [9, 10]. Integration of the various environmental exposures with information on genetic variation through exposomics research can unravel these complex interactions [11] and provide a better understanding of the influences/components contributing to disease or negative health outcomes. Herein we define exposomics [12]as the ensemble of technologies, methodological approaches, and biological research strategies/results that investigate the exposome to characterize exposures, identify biomarkers, and establish mechanistic links between environmental factors and health outcomes. The results of such studies support the development of precision health medicine solutions.

Exposomics research systematically measures and characterizes the impact on health of environmental factors/exposures across the lifespan [13]. Important aspects of this research involve the development of personal exposure monitoring (PEM) systems (comprising sensors, smartphones, geo-referencing, and satellites) for collecting external exposome data at the individual or community levels [14, 15], and analysis of biological samples (that serve as internal markers or biomarkers of external exposures) using multiple -omics technologies [16, 17]. Identification of the relationships between external exposures (as measured by PEM systems {51}and global multi-omics profiles of molecular features in the same individuals constitutes a powerful methodological approach [18] {PMID: 35667843] that opens the way to exposome-wide association studies (EWAS) [19, 20]. The overarching goal of translational exposomics is to use these new tools in risk assessment, and in the estimation of the environmental burden of disease [21, 22], to improve precision prevention and public health intervention strategies [23, 24].

This review aims to provide a clear and compelling rationale for why the exposome matters for genetic research, and how the integration of exposomics can powerfully complement genomic inquiry. Despite the precision and predictive power of genomics, it is increasingly evident that genetic variants alone cannot fully explain disease risk or phenotypic variability. In fact a recent study [25], analyzed data from nearly 500,000 participants in the UK Biobank to assess the relative contributions of genetic and environmental factors–collectively termed the exposome–to mortality and the incidence of common age-related diseases. The study found that environmental factors accounted for 17% of the variation in risk for premature mortality, whereas genetic predisposition contributed less than 2% [25]. The exposome offers a framework to capture environmental and lifestyle influences that interact with the genome to shape health trajectories. As such, this article introduces key concepts, tools, and use cases for exposomics, emphasizing translational strategies that may resonate with geneticists seeking to understand gene-environment interactions, identify modifiable risk factors, or enhance precision health and medicine efforts. The intent is not merely to describe the state of exposomics but to invite researchers from genomics and systems biology to engage with and apply these tools in their investigations. By bridging the conceptual gap between genome and exposome, this manuscript outlines an integrative paradigm that enriches biological discovery and advances personalized medicine and public health.

### Design of exposome studies

Exposome studies are designed to help dissect the “nature versus nurture” conundrum and allow the adoption of a paradigm defined by complex and dynamic interactions between DNA sequence, epigenetic DNA modifications [26], gene expression, metabolic and physiological processes, and environmental factors/exposures that all combine to influence disease phenotypes. Epigenetic changes, such as locus-specific inter-individual DNA methylation differences, arise both *in utero* and after birth [27, 28]. Environmental conditions that can affect the epigenome of an individual include both external and internal factors [29]. Individual lifestyle and behaviors, such as smoking [30], alcohol consumption [31], physical activity [32], diet [33], environmental temperature changes [34], exposure to organochlorine compounds, polychlorinated biphenyls (PCBs), and PFAS [35], stress [36], and viral infections [37], have been shown to have a long-term influence on epigenetic modifications. However, it is possible that small defects in transmitting epigenetic information through successive cell divisions or maintaining it in differentiated cells, accumulate [38] in a process that could be considered as an ‘‘epigenetic drift’’ associated with aging [39]. Indeed, environmental exposures and exposure-modulating factors (such as lifestyle, diet, behavioral and consumer choices, and cultural and socioeconomic or sociodemographic aspects) may have long-lasting effects on metabolism and health, sometimes even in subsequent generations [40]. Knowledge of epigenetic mechanisms (e.g., differential DNA methylation in promoter and intragenic CpG islands as well as in repeated sequences, miRNA expression, skewed X-inactivation, imprinting, chromatin modification) and underlying causes provides a new model for discovering mechanisms affecting disease susceptibility [41]. Last but not least, as a dynamic mediator of environmental interactions, the microbiome (i.e., the collection of microbes living in and on us) [42] can play a pivotal role in modulating how these exposures affect health, especially by influencing metabolism and immune responses [43]. Exposomics operates downstream of genetics [44], and many biologically-relevant sensing pathways likely depend on the activities of gut microbiota and their metabolomic impact [45]. Together, the microbiome and the exposome offer a broader perspective for understanding health. These innovative concepts are essential for advancing precision environmental health [46], i.e. the precise analysis of the link between the state of the environment and human health taking explicitly into account the spatio-temporal nature of human exposures.

Linking external exposure and dose to internal biological responses is crucial [47, 48]. Internal dose resulting from the same external exposure might vary significantly among individuals [49] as the result of differences in physiology (e.g., developmental stage, bodyweight, inhalation rate, obesity status), co-exposure to other compounds (environmental or pharmaceutical), and/or polymorphisms that affect nutrient and xenobiotic metabolism [50].

**Fig. 1:**
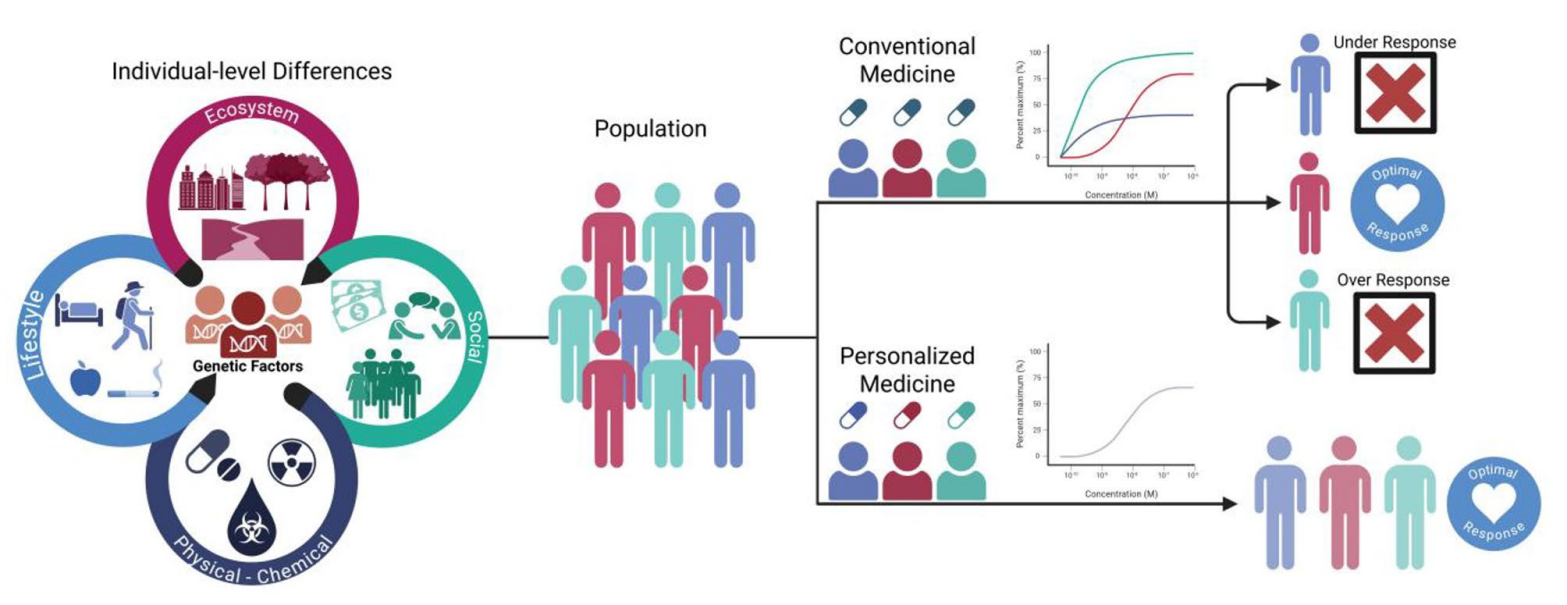
Integrating exposomics and genomics to inform treatment strategies. Environmental exposures (lifestyle, social, chemical, and ecological factors) and genetic factors lead to heterogeneity across populations. In the absence of individualized data, conventional treatment strategies may produce variable responses, with some individuals experiencing suboptimal outcomes. Utilizing exposomic and genomic data to guide treatment strategies can improve efficacy and promote optimal health outcomes by accounting for inter-individual differences

Before conducting genome- and environment-wide association studies [19], it is increasingly recognized that accounting for individual-level differences in internal dose and metabolism can improve the interpretability and precision of results. Tools such as physiology-based biokinetic (PBBK) modeling and profiling of single-nucleotide variants (SNVs) [49, 51] in metabolic genes offer valuable—but not universally applied—approaches to addressing inter-individual variability. While PBBK models have shown promise in research settings and are advancing toward greater regulatory acceptance, their routine use in exposomics remains limited by data availability and model complexity. Likewise, SNV profiling can be informative in studies where metabolic activation or detoxification is central to disease etiology, but may be less relevant when gene-environment interactions do not hinge on specific genetic variants. It is also important to acknowledge that chemical toxicity can arise independently of genetic variation, for example through direct enzyme inhibition or epigenetic modifications [52]. Therefore, these methods are best viewed as complementary tools whose applicability depends on study objectives, available data, and biological plausibility [53]. Figure 1 illustrates a systems-level approach to exposome study design, integrating external environmental exposures, internal dose modeling, multi-omics biomarker analysis, and genomic variation. This framework supports the identification of gene-environment interactions and mechanistic pathways that underpin health outcomes, enabling precision prevention and translational applications.

The collection of human biomonitoring (HBM) data is a key point in the exposome analysis workflow [54]. From these data, bi-directional mechanistic links (external exposure to HBM, and HBM to state of an individual’s health or disease) are investigated in depth according to the approach outlined herein. Misclassification of exposure or its translation into actual biologically effective doses can result in the loss of valuable information [55]. Analysis of the internal exposome (i.e., the ensemble of reactive electrophiles, metals, receptor-binding proteins, endocrine disrupting chemicals, and immune system modulators) needs to be comprehensive [56] because it helps explain how the exogenous molecules identified in HBM samples result in changes in endogenous metabolism [56, 57]. Moving from disease associations to disease causality requires sufficient mechanistic understanding [58] of all intermediate events that promote allostasis, which, in turn, leads to human disease development [59, 60]. Identifying such events comprehensively requires data acquisition (multi-omics in HBM samples, with a special focus on transcriptomics, proteomics, and metabolomics) and data interpretation that includes regulatory and pathway analyses [61, 62]. It is also important to highlight the contribution of cumulative exposure to health stressors and related modifiers to the burden of human disease [63, 64]. Agnostic transcriptome and metabolome analyses on biological samples [65] and subsequent joint pathway analyses [66] result in the identification of molecular signatures that have the potential to serve as surrogates for exposure biomarkers [62, 67, 68]. Even when exposure to single compounds only shows moderate adverse effects, it has been found that co-exposure to a real-life mixture [67] may have supra-additive effects [69] on gene expression modulation [70]. After the agnostic tier of analysis, it is possible to identify not only single genes that have shown significant modulation in expression levels [71], but also the biological pathways that are regulated by gene networks that were significantly modulated [72]. When combined with the changes in metabolomics profiles [73], joint pathway analysis can reveal the key pathways involved in each perturbation (e.g., p53 or oxidative stress) [74], and how these are differentially modulated by specific chemical families; specific genes, gene sequences, and combinations of other small molecules could then be characterized as molecular markers of exposure [75]. By further proceeding with targeted analysis focusing on the adverse health outcome pathways identified agnostically [76], causal relationships among genome, environment (including dietary, consumer and lifestyle choices) and specific effect biomarkers or disease phenotypes can be deciphered [77].

### Examples of exposome studies

To concretely illustrate how exposomics can address complex health questions that are difficult or impossible to answer through traditional approaches, we present two detailed case studies. These examples walk the reader through the design, implementation, and interpretation of exposome-based research, highlighting each step of the process — from environmental and biological sample collection to omics data integration and causal inference. They show how the exposome paradigm enables not only comprehensive exposure assessment but also mechanistic insight and targeted risk prediction, ultimately guiding more effective public health action.

One illustrative example of the exposome paradigm in action is the HERACLES study, which investigated long-term exposure to a major waste landfill in Athens (Fili landfill) and neurodevelopment in children. The Fili landfill is one of the largest in Europe, receiving ~ 6,000 tons of solid waste per day (primarily organics, paper, and plastic)​. Even though only non-hazardous municipal waste is officially deposited there, the adjacent older landfill (Ano Liosia) had received industrial hazardous waste in the past, leaving a legacy of soil contamination​. To capture the full scope of exposures in this setting, HERACLES recruited approximately 325–350 children (ages 3–8) living within 0.5–12 km of the landfill​. These children have been followed since 2012 with an exposome-based design: the research team measured environmental contamination (e.g. heavy metal concentrations in soil at the child’s residence), conducted human biomonitoring for pollutants (levels of arsenic, cadmium, and mercury in the child’s urine; lead in blood; manganese and mercury in hair) as objective indicators of exposure, and recorded additional proxies like residential distance from the landfill​. In parallel, extensive data on diet and lifestyle were gathered– detailed information on each child’s food consumption (meat, fish, dairy, fruits, etc.), breastfeeding history, body size, and family socioeconomic status (parents’ education, income) and psychosocial stressors​. Importantly, the study also included cutting-edge metabolomics: analysis of metabolic profiles in children’s urine with pathway analysis to identify biochemical pathways perturbed by the exposures​. Child neurodevelopment was repeatedly assessed using standardized cognitive tests (WISC-IV intelligence scale)​, and all these variables were analyzed in concert using an exposome-wide association approach.

The exposome analysis confirmed that proximity to the waste site– and the associated exposure to landfill-derived pollutants– has a measurable impact on children’s neurodevelopment [78]. Children living closer to the landfill (where higher concentrations of toxic metals were detected in soil) tended to score lower on cognitive developmental tests, indicating a detriment to neurodevelopmental progress​. In fact, soil heavy metal levels showed a strong inverse gradient with distance (the closer to the contaminated site, the higher the metal levels), which was reflected in poorer neurodevelopmental outcomes in nearby children​. These results implicate chronic exposure to landfill-related contaminants (such as heavy metals) as a critical risk factor for neurodevelopmental delays. Conventional analyses have long suspected such associations (e.g. lead exposure and IQ loss), but the exposome framework provided deeper insights by simultaneously evaluating numerous other factors that modulate or confound this relationship. Notably, the HERACLES study found that socioeconomic and lifestyle factors significantly modified the effect of environmental exposures on neurodevelopment​. For example, parental education emerged as a protective factor: children with more educated parents had higher IQ scores on average, even after accounting for pollution exposure. In the exposome-wide analysis, parental education level was in fact one of the strongest predictors of child IQ (aside from direct contaminant measures), with mother’s and father’s education each showing a positive association (β ≈ 0.30 increase in IQ score per higher education level, *p* ~ 10^− 7^)​. Family socioeconomic status (SES) similarly showed a beneficial effect​. These findings suggest that a stimulating, resource-rich home environment can partly buffer the neurodevelopmental harm caused by toxic exposures.

A second case exemplifies how the exposome paradigm can address acute exposure events and latent health risks. In 2015, a major fire broke out at a plastics recycling plant in the Aspropyrgos area of Athens, releasing a dense plume of smoke over surrounding residential areas. Under normal operation, this recycling facility had been considered relatively benign– exposomic assessment indicated that proximity to the plant did not pose significant health threats to the community​. However, the accidental fire dramatically changed the exposure scenario. Burning of mixed plastics can generate a cocktail of hazardous compounds, most notably polychlorinated dibenzodioxins and furans (PCDD/Fs), which are persistent organic pollutants with potent toxic and carcinogenic properties​. During the Aspropyrgos fire, large quantities of these compounds were released over a short period. Ambient air measurements taken around the 5th day of the fire showed dioxin toxic equivalent (TEQ) levels of about 1.8 pg/m³ (WHO-TEQ) in the nearby community– on the order of 25 times higher than typical background levels in industrial areas of Athens (~ 0.1 pg/m³), and comparable to the extreme concentrations seen in severe waste fires​. Simply put, the population experienced in a few days an exposure that would normally accrue over years. Traditional risk assessment might treat this as a brief, transient spike– significant for acute toxicity, but not necessarily for long-term risk (since the exposure lasted only 5–6 days). The exposome approach, by contrast, recognizes that certain chemicals like dioxins bioaccumulate and persist in human tissues, effectively extending the exposure internally well beyond the fire event itself [79]. To evaluate the long-term health impact of this acute incident on children, researchers applied a comprehensive methodology (the INTEGRA exposome framework​) that integrated environmental measurements, exposure modeling, internal dose kinetics, and biomonitoring data. First, they reconstructed the population’s dioxin exposure profile using both monitoring data and modeling. Importantly, instead of assuming the risk could be calculated from just a few days of inhalation dose, they used a physiologically based biokinetic (PBBK) model to simulate how dioxins would distribute, accumulate, and clear in the human body over time​. This yielded an internal dose metric– the area-under-the-curve (AUC) of blood dioxin concentration over ensuing years– as a more realistic measure of biologically relevant exposure​. The model was calibrated with background biomonitoring data (prior measurements of dioxin levels in Athens residents) to ensure it reflected typical pre-fire exposure​. Modeling results showed that even a one-time exposure to the fire’s dioxin plume could elevate dioxin body burdens for decades. The average blood concentration of PCDD/Fs in the exposed population was projected to climb to ~ 18 pg TEQ per gram of lipid shortly after the fire (compared to ~ 7 pg/g lipid before the event)​. This ~ 2.5-fold increase in dioxin load would not rapidly disappear; due to dioxins’ persistence, modeled blood levels remained above the original baseline for many years following the fire​. Thus, the short-term inhalation translated into a long-term internal exposure. The implications were especially concerning for infants and unborn children. Exposome assessment showed that if a mother was exposed to the fire, the dioxins in her blood could be transferred to a fetus during pregnancy and to an infant via breast milk. Fetuses have a high proportion of body fat; thus, they can sequester a higher proportion of lipophilic toxins. The model indicated that an exposed mother would indeed deliver dioxins to her fetus such that fetal blood concentrations would mirror the mother’s, resulting in an estimated 17% higher cumulative dioxin exposure (AUC) over the child’s lifetime than if the fire had not occurred​. Likewise, breastfeeding by an exposed mother was predicted to significantly add to an infant’s dioxin intake: the concentration in breast milk could reach ~ 10 pg/g lipid​, leading to an overall lifetime exposure increase of about 22% for breastfed infants in the affected area​. These findings highlight how early-life exposures, even indirect and brief, can have disproportionate effects on a child’s long-term chemical burden– a result that standard risk calculations (focusing on adult exposure duration) would overlook​. Accounting for the increased lifelong dose in infants (in utero + breast milk exposure), the model projected an ~ 18% increase in lifetime cancer risk for a child exposed in utero, and up to ~ 22% increase if that child was also breastfed after the fire​. Thus, exposome analysis identified breastfeeding infants of exposed mothers as a particularly vulnerable group, for whom the fire could have a lasting carcinogenic impact. Conventionally, such specific risk elevation might not be recognized, since infants were not directly breathing the smoke (the exposure came via maternal transfer), underscoring the importance of the holistic exposure accounting.

An integral part of the exposome paradigm is verifying and deepening these modeling predictions with empirical data and biological markers. Following the Aspropyrgos fire, biomonitoring of local residents was conducted, and it indeed confirmed elevated internal exposures as the model had suggested. Blood samples from people living near the plant showed PCDD/F levels of ~ 12.4 pg/g lipid– significantly higher than the ~ 7.4 pg/g lipid background level in unexposed populations​. This measured increase (≈ 5 pg/g) in community dioxin burden is consistent with the model projections for the impact of the fire​. Moreover, by applying untargeted metabolomics (just as in the HERACLES study), early biological effects related to this exposure could be detected. Comparisons of blood metabolite profiles between fire-exposed individuals and unexposed controls revealed a shift in lipid metabolism: exposed individuals had higher levels of unsaturated fatty acids relative to saturated fatty acids in their blood​. This pattern is a biochemical fingerprint of altered cholesterol and lipid homeostasis. It aligns with the known mechanistic action of dioxins via the aryl hydrocarbon receptor (AHR) pathway. Dioxins bind to the AhR, a transcription factor, which not only triggers detoxification enzymes (like CYP1A1) but also interferes with lipid metabolism regulation​. Exposome-derived metabolomic data indicated that such AhR-mediated pathway disruption was occurring: the increase in unsaturated fatty acids suggests perturbation of cholesterol biosynthesis, an effect linked to AhR deregulation of sterol regulatory element-binding proteins​. In short, the exposome approach was able to capture a molecular signature of the exposure (altered fatty acid profiles) that points toward a causal pathway (AHR signaling and downstream metabolic changes) leading to potential health outcomes (e.g. elevated cancer risk, given the role of AHR in tumor promotion).

This mechanistic evidence strengthens the case that the fire-related contamination did biologically affect residents, beyond what epidemiological statistics alone could show. It is an edifying demonstration of how environmental monitoring, internal dose modeling, and multi-omics biomarker analysis can be woven together in an exposome framework to assess an acute ICS event. By comparison, a conventional chemical risk assessment might have simply noted that dioxin emissions exceeded safe limits and perhaps estimated a generic excess cancer risk for the population. The exposome paradigm went further– it identified who among the population (infants, in this case) would carry the highest risks, quantified how long those risks would persist (through persistent body burdens), and even revealed the early biological perturbations happening in their bodies. Such insights are invaluable for public health decision-making, for example by justifying targeted health monitoring of infants born to exposed mothers, or by emphasizing the need for rapid soil and food-chain decontamination after the fire (since dioxins deposit and linger in the local environment).

Together, the case studies described in these papers underscore what is *unique and advantageous* about the exposome paradigm in evaluating hazardous waste and industrially contaminated site impacts (ICS) on children. Unlike conventional methods in environmental epidemiology and chemical risk assessment, the exposome framework integrates multiple layers of information (environmental, biological, and social) to build a more complete exposure-health association. By doing so, it not only quantifies risk more accurately but also illuminates the underlying causes of that risk. This has practical implications for risk management. For instance, finding that neurodevelopmental outcomes were linked to both metal exposure and nutrition suggests that remediating a contaminated site and improving community nutrition could synergistically benefit child health. Detecting a specific metabolic pathway disrupted by pollution (e.g. the cholesterol biosynthesis pathway by dioxins, or the mevalonate pathway by metals) provides molecular targets for further research and sometimes even potential biomarkers for early diagnosis or intervention. In short, the exposome approach enables a form of precision public health or *precision prevention*: it helps identify the combinations of exposures and susceptibilities that put certain children at higher risk, and thus allows interventions to be directed where they will be most effective.

The key advantages of using the exposome paradigm for children living around contaminated sites, are summarized as follows:

- *Comprehensive exposure integration*. The exposome paradigm assesses multi-factorial exposures as a whole. Children are rarely exposed to one chemical at a time; an exposome study can evaluate multiple chemical and non-chemical stressors together, mapping out an “expotype” that reflects real-life complexity. This reveals combined effects (e.g. co-exposure to heavy metals, airborne particulates, organic toxins, along with lifestyle and social determinants) that conventional single-substance studies would miss.
- *Internal dosimetry and biomonitoring*. Exposome research tracks how much of a toxicant actually gets into a child’s body– for example via blood or urine biomarkers– and employs toxicokinetic modeling to understand retention and timing​. This is crucial for substances like dioxins with long half-lives. Traditional assessments based only on external concentrations (e.g. ambient air levels) can severely underestimate or mischaracterize risk for bioaccumulative toxins. The exposome focus on internal dose provides a more biologically relevant risk estimate.
- *Mechanistic insights through -omics*. Incorporating high-throughput -omics (exposomics, metabolomics, transcriptomics, epigenetics, etc.) the exposome approach identifies early biological changes caused by exposure. These mechanistic data (such as altered metabolic pathways or gene expression profiles) bridge the gap between environmental exposure and disease. In the studies above, omics data highlighted plausible pathways for neurodevelopmental toxicity (oxidative stress, cholesterol metabolism) and carcinogenesis (AhR signaling), lending support to causal interpretations. Conventional epidemiology rarely captures such mechanistic evidence.
- *Contextual and sociodemographic factors*. The exposome framework explicitly incorporates factors like socioeconomic status, education, stress, and diet as part of exposure assessment. Rather than treating these as mere confounders to adjust away, exposome studies treat them as integral components of exposure– often as effect modifiers or co-exposures. Accounting for such interactions leads to a more refined understanding of risk and resilience in communities near ICS.
- *Enhanced causal inferences*. By linking external exposures to internal doses to molecular effects to clinical outcomes, the exposome paradigm provides a chain of evidence that greatly strengthens causal inferences.​ Such triangulation of evidence (epidemiological, toxicological, and mechanistic) is a major advantage over conventional studies that often rely on correlation alone. This comprehensive evidence base enables targeted actions– for example, focusing remediation on specific contaminants or tailoring public health advice (like encouraging diets rich in omega-3 for exposed populations).

The exposome paradigm represents a transformative step forward in how we evaluate the health impacts of hazardous waste and industrial contamination on children. By integrating environmental measurements, internal biological markers, and socio-economic context, it provides a holistic view of exposure that can identify hidden risk factors and protective factors. The examples from the outskirts of Athens, Greece demonstrate that an exposome approach can both detect harm (confirming that living near a toxic landfill can impair child development, or that a brief toxic emission can raise long-term cancer risk) and explain it (by uncovering the pathways and conditions that determine that harm). This depth of understanding is exactly what is needed to design effective interventions. Using exposomics-derived evidence public health officials and communities can move from broad associations to targeted solutions– such as reducing specific pollutants, improving nutrition, or providing social support. Ultimately, the exposome paradigm aims to enable precision prevention and optimized risk management in ICS-affected areas​, ensuring that we protect those children who need it most with strategies grounded in a thorough scientific understanding of their world. Hence, the complexity of industrial contamination impact on health can be met with an equally comprehensive research paradigm, yielding insights that translate into healthier futures for children.

While the exposome framework alone provided rich insights into the environmental drivers of health outcomes in these examples, the addition of genomic data could further enhance their interpretative power. For instance, in the HERACLES study, incorporating genetic polymorphisms related to metal metabolism or neurodevelopmental pathways (e.g., APOE, BDNF) might help explain differential susceptibility among children. Similarly, in the Aspropyrgos fire case, examining genetic variation in detoxification enzymes (such as CYP450s, GSTs, and ALDHs) or in dioxin receptor pathways (e.g., AHR) could clarify why certain individuals exhibited more pronounced metabolic changes. Conversely, exposomic data can help contextualize genomic associations by identifying modifiable environmental triggers that interact with genetic predisposition. This bidirectional integration—of genome informing exposome response, and exposome refining genomic risk—embodies the promise of precision environmental health and precision medicine. Future studies that combine these data layers will enable deeper mechanistic understanding, more accurate risk stratification, and ultimately, more personalized and effective prevention strategies.

### Integrative platform for exposomics

**Fig. 2:**
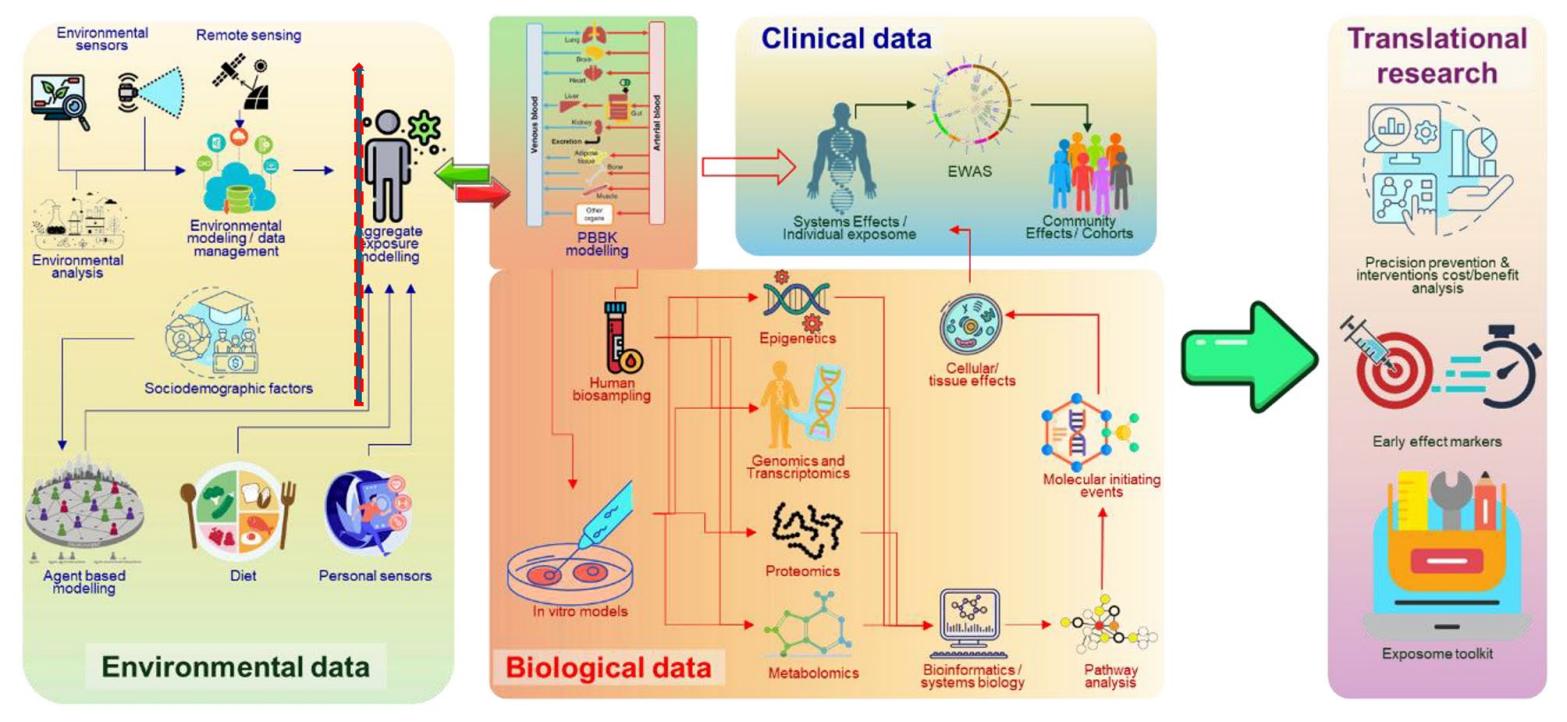
Concept of an exposomics platform. The green arrow indicates that we can move from internal exposure data to external exposure data, the red arrow indicates the translation of aggregate and cumulative external exposure to internal exposure; PBBK modelling is the (bidirectional) link between external and internal exposure. Personal sensors contribute to exposure assessment, and they can provide information related to ancillary exposure data as well (i.e. heart rate, intensity of activity, location etc.), not only environmental exposure data. The upper line includes also monitoring and chemical analysis data, but all of them related to environment

The ideal exposomics platform (Fig. 2) brings together and organizes environmental exposures, demographics, socio-economic factors, biomarkers, and health outcome data using a discovery- and data-driven paradigm [20, 80–82]. This platform includes multi-omics and advanced bioinformatics approaches [74, 83] that couple data mining with systems biology and physiology-based biokinetic (PBBK) and exposure modeling, to ensure that environmental exposure-health associations are analyzed comprehensively following the adverse outcome pathway paradigm [84]. The overall approach needs to be verified at the community level and in population studies [85, 86] to permit analysis of the impact of various levels of environmental exposure, age windows and gender differentiation of exposure, and socio-economic and genetic variability. This enables a detailed evaluation of the link between exposomics and genomics [87, 88] and its translation into a comprehensive assessment of the overall burden of disease in humans. The latter can then be used for developing precision prevention and intervention strategies, identifying early effect biomarkers, and, finally, putting together an exposome toolkit to facilitate the uptake of exposome-based evidence into public health policy development.

Novel tools are essential for exposome research [20, 89] to permit complex environmental health challenges to be addressed [90, 91]. Examples include co-exposure to ultrafine particles and bioallergens [92], emergence of environmental chemicals in water and soil matrices stemming from hazardous waste and industrially contaminated sites [93], microplastics and per- and polyfluoroalkyl substances (PFAS) in water bodies, as well as co-exposure to chemicals of emerging concern in consumer goods and food residues [94]. Critical to the success of these investigations will be the ability to bring together and harmonize existing geospatial, environmental, health and socioeconomic data, and to collect new high-resolution data using innovative environmental micro-sensors, remote sensing or other community and -omics/systems biology-based approaches. Such data can be used, for instance, to describe the relationship of the exposome to endocrine disruption [95, 96] and sex-related changes, e.g., menopause [96]. Ultimately, exposome evidence coupled with genomics data enables us to explain the risk and actual burden of diseases, such as cancer [97–99], neurodevelopmental [100, 101], neurodegenerative [102, 103] or cardiorespiratory and metabolic diseases [104, 105], among others. In this context it is critical to identify windows of susceptibility throughout the entire lifespan [106], including from preconception through puberty, adolescence and adulthood [107]. Such research also involves assessing whether the burden of diseases influenced by environmental factors is distributed equally among populations or exerting an overt influence in select vulnerable populations [108], such as gender and ethnic minorities [109], socio-economically disadvantaged individuals [110], or those living in regions with known high contamination and pollution [111].

Mapping environmental exposures through the entire lifecycle of an individual, while ideal, may not always be necessary, such as in cases where critical lifetime events of an individual’s geospatial lifeline cross a noteworthy exposure event [112] that is recognized and understood. Hence, exposure episodes with the largest impact on drawing the exposome profile in an individual’s life could be reconstructed and linked to socio-economic conditions at critical life stages, such as pregnancy, puberty, the reproductive age period and menopause (for women) or the 50–55 years age window (for men). Modeling the space-time trajectories of the at-risk population at the individual level is challenging. Indeed, considerable conceptual and computational difficulties have been encountered when intersecting data on the distributions of pollutants with the patterns of movements of exposed individuals or groups; this is primarily due to the limitations of available data on environmental conditions and human distributions. However, with the advent of geographic information systems (GIS), GPS to track individuals, and personal environmental monitoring, the undertaking of such analyses throughout an individual’s lifetime is now possible.

### Causality in exposome-health associations

Causal exposure-response associations can be drawn [113] with explicit consideration of variations in both the individual exposome [114] and genetic patterns in populations under study. This serves as a foundation of solid scientific evidence upon which to design interventions that foster precision prevention and promote public health in different environmental settings [115]. The exposome introduces a new paradigm for interdisciplinary scientific work in environment and health [20, 68, 116]. It represents an approach that builds upon the exploration of the interconnections between the co-existence of multiple stressors [117] and the different scales of biological organization [118] that, together, produce the final adverse health outcome [119]. This approach marks a clear departure from the conventional paradigm [120] that seeks to shed light on the identification of singular cause-effect relationships between stressors and health outcomes [121]. It entails creating a new way of combining health-relevant information coming from different disciplines, including (but not limited to) environmental science, epidemiology, toxicology, physiology, molecular biology, biochemistry, mathematics and computer science [22, 116]. In a truly exposome-based approach, all factors affecting the internal and external exposome are treated as co-variates that define the exposome, rather than just as confounders [73]. Functional integration of these different information classes into a unique framework will result in understanding the complex interaction between the genome and environmental exposures [122]. Indeed, exposomics can lead to interventions, policies, and practical applications [123] in precision prevention and medicine and, in so doing, promote the development of a precision environmental health paradigm [124, 125].

Assessing exposomic alongside genetic data can help identify specific environmental exposures that increase disease risk in individuals or populations [126], thereby enabling personalized interventions. For example, research has shown that approximately one-third of breast cancer cases cannot be attributed to genetic factors alone, suggesting a significant environmental component [127]. Exposome studies have identified several environmental factors associated with an increased risk of breast cancer, including chemical exposures (dioxins, DDT, PFOSA), air pollution, and occupational exposures to solvents, gasoline components, and other mammary carcinogens. In addition, air pollution as a trigger for asthma can guide targeted efforts to reduce exposure in predisposed communities [128, 129]. Exposome research has revealed that low-level chronic exposure to hazardous waste may have adverse effects on children’s neurological and congnitive development, in combination with specific diets and low maternal educational level [78].

Understanding how environmental exposures affect gene expression and lead to epigenetic changes [130] allows for more personalized treatments. Studies linking environmental exposure factors to diseases, such as type 2 diabetes, have emphasized the need for exposome data in disease-risk prediction and treatment customization, especially across diverse populations [129, 131].

### Translating exposome research results for precision prevention

Translational science linked to exposomics can inform public health policies, enabling population-level risk reduction and relevant regulatory action [132]. Exposomics identifies environmental risks affecting specific populations, thereby allowing policymakers to target high-risk groups or areas through the development and implementation of tailored regulations that take into account the multiple factors contributing to the health risk [133]. For example, pollutants linked to cancer can lead to stricter regulations in affected regions, while considering the socioeconomic and cultural or dietary patterns that modulate the link between the exposome and carcinogenesis. Exposome data can inform policies on chemical exposure [134], air quality [135], and workplace safety [136], especially for high-risk industries like agriculture, manufacturing, and waste management [78, 134, 137].

Practical applications in precision prevention [138] include the development of preventative strategies by identifying harmful exposures based on genetic profiles [139]. Early warnings about multiple emerging pollutants (e.g., ultrafine particles; biologicals, including microorganisms, allergens, and other biological matter such as microbial volatile organic compounds and mycotoxins; desert dust) coupled with infective pathogens [140] can lead to preventive actions like using air purifiers [141, 142] or inciting vulnerable populations to avoid specific polluted areas [143]. The recent COVID-19 pandemic and its dire public health effects highlight the importance of this integrative translational research approach. Exposomics data can also support personalized lifestyle recommendations, such as dietary changes to reduce exposure to harmful substances [144] or minimize time spent in polluted environments [78]. On the community level [145], exposomics data can shape healthier urban environments [146], reducing air pollution and noise [147] through better waste management [148], creation and maintenance of green [149] and blue [150] infrastructure, and development of transportation networks [151, 152]. Research on the exposome can inform public health campaigns that raise awareness about environmental risks [153] and promote safer behaviors [154], and empower communities to advocate for health protections [145].

Finally, multi-omics-based exposomics analyses [17, 101] offer a more expanded, objective view of individual health risks, enabling precision prevention and more accurate and personalized risk assessment [155]. As a comprehensive approach, they improve predictive models for chronic diseases by accounting for cumulative exposures [156]. Moreover, machine learning applied to large exposomics datasets [74, 83] helps find individuals most at risk for conditions like cancer or cardiovascular diseases, facilitating personalized preventive strategies.

## Conclusions

The human genome, while offering insights into disease, only tells part of the story. Scientists have introduced the concept of the “exposome”—the sum of all environmental exposures an individual experiences throughout their life—as a driver of health and disease that complements the genome. Understanding the exposome, and how it interacts with our genes, is key to preventing and treating many diseases and enhancing precision health and medicine approaches.

Researchers are developing sophisticated methods to measure the exposome, from using sensors to track environmental exposures to analyzing our body’s molecular responses (multi-omics). This involves creating detailed profiles that capture the complex interplay between external exposures and internal biological responses. By combining these exposome profiles with our genetic information, scientists aim to understand the mechanisms through which environmental factors affect health. This is not just about identifying links; it is about understanding cause-and-effect relationships. This involves sophisticated modeling techniques (including physiology-based models to translate external exposures/factors to internal biological impact) and advanced computational methods (e.g., machine learning and AI tools to analyze large, complex datasets). The approach also takes into account the crucial role of epigenetics (i.e., changes in gene expression that are not caused by changes in DNA sequence), proteins, lipids, and other small molecules, and the microbiome in how an individual responds to environmental exposures.

By identifying specific environmental factors and exposures that increase adverse health outcome risk in individuals, the development of improved prevention strategies and targeted interventions at the individual and population levels is thus feasible. This might manifest as advocacy for better air quality in polluted areas, personalized dietary recommendations based on individual genetic and environmental profiles, or even new treatments for diseases that manifest, in large part, by environmental exposures. The potential to improve public health and design preventative measures for individuals and populations alike is vast. This new, multidisciplinary approach promises to revolutionize how we prevent and treat human disease.

## Data Availability

No datasets were generated or analysed during the current study.
